# Decreasing boarders in the emergency department by reducing clerical work in the discharge process of in-hospital patients in Brazil – an interrupted time-series analysis

**DOI:** 10.1186/s12873-022-00656-y

**Published:** 2022-06-07

**Authors:** Diego Marques Moroço, Antonio Pazin-Filho

**Affiliations:** 1 Ribeirão Preto Medical School, University of São Paulo, Ribeirão, Brazil; 2 Department Ribeirão Preto Medical School, University of São Paulo, Ribeirão, Brazil

**Keywords:** Patient discharge, Length of stay, Six sigma and lean, Hospital quality, Electronic health records, Digital signature, Business process management, Interrupted time series outpatient clinics, Hospital, Crowding, Emergency department

## Abstract

**Background:**

Emergency Department (ED) boarding is related to in-hospital patients' discharge since no beds will be available for receiving ED patients if there is a delay for patients in the yard leaving the hospital. New techniques implemented in hospital institutions, such as digital signatures to facilitate clerical work improve these processes. We evaluated the impact of expediting patients' discharge after medical orders with the number of patients with an unplanned hospital admission from the Hospital Out Clinic directed to ED for waiting for an available bed in a public tertiary hospital in Brazil.

**Methods:**

We conducted a quasi-experimental study before and after an intervention. It consisted of an encrypted digital signature to reduce clerical work and expedite the patient's release from the institution after medical discharge. We used an interrupted time-series analysis based on administrative data (number of hospital discharges, bed turnover, the time between medical discharge, and the time the patient effectively left the hospital) from 2011 to 2020.

**Results:**

We enrolled 210,496 patients admitted to the hospital from January 2011 to December 2020. Of those, 69,897(33%) composed the group after the intervention. There was no difference between the groups' gender, age distribution, the proportion of surgical patients, or in-hospital stay (≤ 7 or > 7 days). The interrupted time series analysis for the time from medical order to effectively hospital discharge showed an immediate change in level (Coefficient β2 -3.6 h—95% confidence interval -3.9;-3.4), but no a difference in the slope of the behavior of the post-intervention curve (β3 0.0005 coefficient—95% confidence interval -0.0040;0.0050). For the number of patients directed to ED, we observed no immediate change in level (Coefficient β2 -0.84 patients—95% confidence interval -0.33;0.16), but a difference in the slope of the behavior of the post-intervention curve (β3 0.0005 coefficient—95% confidence interval -0.0040;0.0050).

**Conclusion:**

Reducing clerical work and expediting patient discharge was associated with decreased potential boarders to ED.

## Background

Emergency Department (ED) boarding is the output of several processes [1]. One of these is Hospital Out Clinic (HOC) patients who need an unscheduled hospital admission during their visits. If there are no beds available when the hospital admission is requested, the patient is directed to ED to wait and start treatment [2, 3]. Emergency Department (ED) boarding is related to in-hospital patients' discharge since no new available be will occur for receiving ED patients if there is a delay for patients in the yard leaving the hospital.

Our institution is a Brazilian tertiary referral public teaching hospital with 4,5 million inhabitants in the northeast region of Sao Paulo. We have around 3,000 external visits (HOC) every weekday, and a common problem is unscheduled hospital admission directed to ED [4].

Our hospital implemented an in-house developed Electronic Health Record (EHR), which continuously actualizes and allows us to customize processes. We use business process management (BPM) tools to implement any change in the EHR and succeed in other areas of study as sepse [5]. We recently reviewed the discharge process using a through output method [5]. We realized that the physician's role in the first steps of the process involves a high degree of variability, considering the diversity of teams and our institution's teaching characteristics.

Otherwise, the patient discharge after medical order was the final step and took seven hours on average, mainly due to clerical work required by Brazilian regulations. We hypothesized that reducing this time would be possible using a nurse encrypted digital signature (DS) as a trigger in the EHR to allow safe discharge. Changing this step would also imply more beds available sooner, avoiding directing patients to ED. Besides, it would interrupt several procedures, such as sending meals and medications to the floor after the patient has left the hospital.

## Methods

We conducted a quasi-experimental study before and after an intervention to expedite the patient's release from the institution after medical discharge. We performed the analysis based on administrative data (number of hospital discharges, bed turnover, the time between medical discharge, and time the patient effectively left the hospital) from January 1, 2011, to December 31, 2020.

We used interrupted time-series analysis (ITS) to evaluate the discharge process intervention after medical order in the direction of unscheduled hospital admissions to the ED. We extract the data from our Electronic Medical Record (EMR), provided by the Clinical Hospital of the Ribeirão Preto Medical School of the University of São Paulo – Brazil. Our institution's Ethical Committee approved the study (CAE 96,424,318.2.0000.5440).

**Fig. 1:**
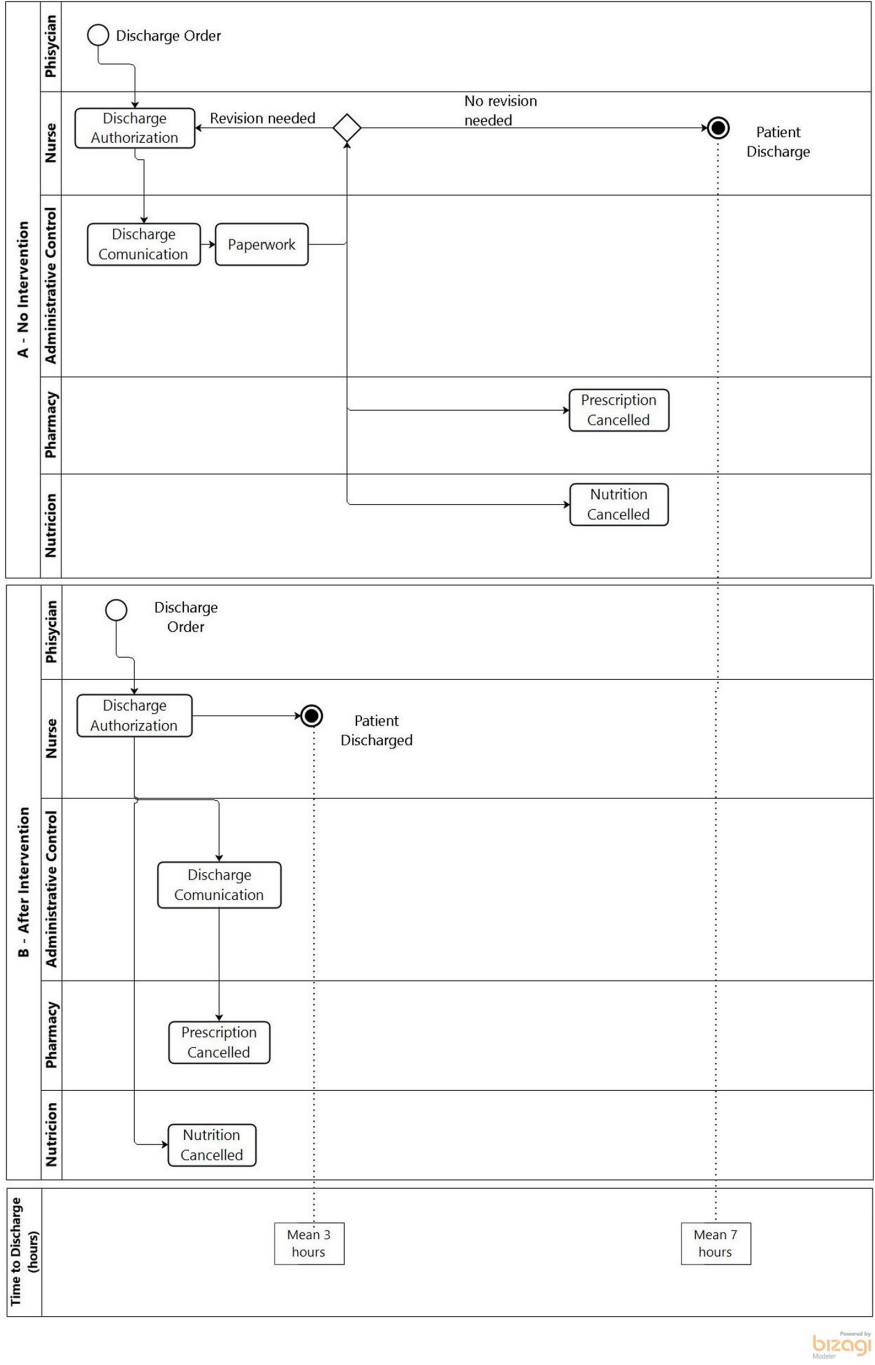
Discharge process after medical order before **A** and after **B** the intervention.

We designed the BPM current process before (Fig. 1A) and after (Fig. 1B) the intervention implementation on January 1, 2017, using Bizagi®. The intervention consisted of simplifying the clerical work. We used a nurse-encrypted digital signature (DS) to trigger canceling prescriptions and nutrition to be sent to the floor and inform the administrative control that the patient had left the hospital. The nurse also documented any medications provided to the patient. The encrypted DS introduced in the EHR on January 1, 2017, guaranteed safe discharge.

We chose to include data from 2011 onwards since our EHR started to function that year. We performed a sensitivity analysis reducing the time for three years prior to and post-intervention, and there was no change in the results. Based on that and the consistency of the nurse discharge process after the medical order in our institution, we decided to keep the baseline results. We excluded data after December 31, 2020, due to our institution's COVID-19 (2019 Novel Coronavirus Disease) pandemic changes [6]. Our models hypothesized that the intervention would be immediate and sustained in time. Since the duration of in-hospital stay correlates with different outcomes, we first performed the ITS analysis for the whole group and stratified it into two groups (≤ 7 or > 7 days).

We also recorded the average number of daily patients directed to ED by month during the same study time and treated them equally with ITS to evaluate the intervention's impact.

We used the itsa command routine in Stata 15 ® to conduct ITS analysis and construct graphics. The itsa command routine estimates the parameters according to equation Y_t = β_0 + β_1 T + β_2 X_t + β_3 TX_t + _t. It measured the aggregated outcome variable at each equally spaced time point t, T is the time since the start of the study, Xt is an indicator variable representing the intervention, and TX_t is an interaction term. In a single-group study, β0 represents the intercept or starting level of the outcome variable. β1 is the slope or trajectory of the outcome variable until the introduction of the intervention. β2 represents the change in the outcome level immediately following the intervention's introduction. β3 represents the difference between preintervention and postintervention slopes of the outcome. Thus we look for significant *p*-values in β2 to indicate an immediate treatment effect and in β3 to demonstrate a treatment effect over time. Comparing groups follows the same rules but adds interaction terms [7].

## Results

**Fig. 2:**
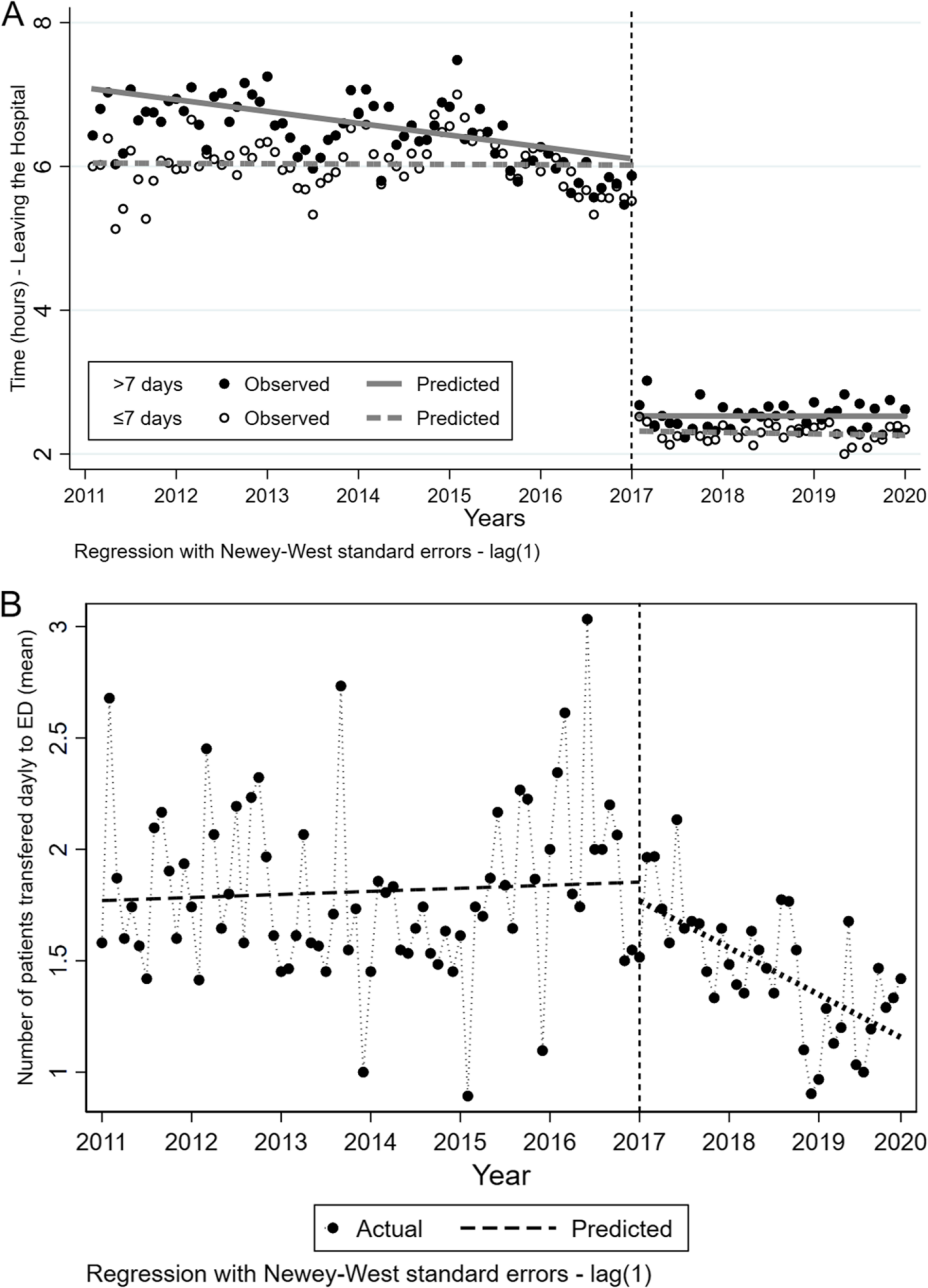
Interrupted Time-Series analysis of time to discharge after medical order **A** and mean of directed patients to the Emergency Department **B** before and after the intervention – *p* < 0.05 for both scenarios

We enrolled 210,496 patients admitted to the hospital from January 2011 to December 2020. Of those, 69,897 (33%) patients composed the group after the intervention. There was no difference between the groups' gender, age distribution, the proportion of surgical patients, or in-hospital stay (≤ 7 or > 7 days). The interrupted time series analysis for the time from medical order to virtually hospital discharge showed an immediate change in level (Coefficient β2 -3.6 h—95% confidence interval -3.9; -3.4—Lag1), but not a difference in the slope of the behavior of the post-intervention curve (β3 0.0005 coefficient—95% confidence interval -0.0040; 0.0050—Lag1). There was no change in the results with stratification by in-hospital stay (Fig. 2A).

For the number of directed patients to ED, we observed no immediate change in level (Coefficient β2 -0.84 patients—95% confidence interval -0.33; 0.16—Lag1), but a difference in the slope of the behavior of the post-intervention curve (β3 0.0005 coefficient—95% confidence interval -0.0040; 0.0050—Lag1) – Fig. 2B.

**Fig. 3:**
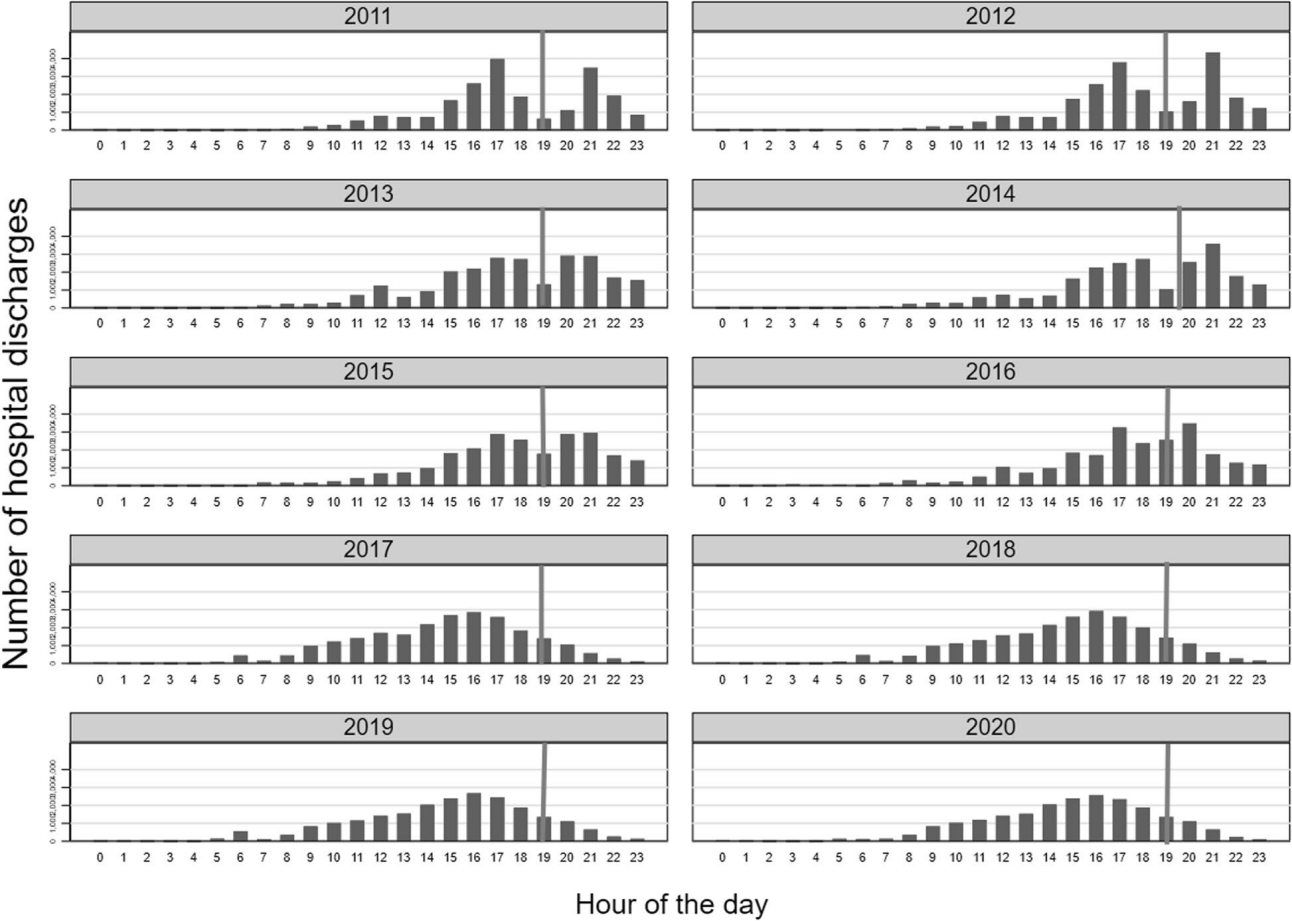
Histograms for the number of patients discharged accordingly to the hour of the day by year of study. The line marks the nurse duty shift at 19h00

In histograms, we presented the number of patients discharged by the day's hour (Fig. 3). Before the intervention in 2017, there was a bimodal distribution, with a considerable amount of discharges occurring after 19h00 (2011–2016), the time to nurse shift change in our institution. After the intervention, we can see a distribution shifted to the left, with most discharges occurring between 15h00 and 16h00 (2017–2020). Furthermore, the distribution now resembled a Normal distribution.

## Discussion

We showed that a simple intervention based on an encrypted digital signature as a trigger of events in an EHR could reduce the time to discharge after medical order around a median of 3.6 h by reducing clerical work. Furthermore, this change was immediate and sustained after the intervention. Finally, we showed a slight and persistent decrease in the number of patients directed to ED waiting for an available bed on the floor.

Reducing boarders in the ED is a complex problem. The phenomenon is associated with several factors and different intensities depending on the institution studied. Therefore, identifying the leading causes to solve is paramount. In our institution, unscheduled hospital admission indicated to HOC patients was a workable BPM evaluation problem. According to Brazilian legislation regarding clerical work, the main problem was guaranteeing safe discharge after medical order [8]. Before 2017, Brazilian law obliged healthcare personnel to ensure that everything was correct before the patient left the hospital. Even though we have an EHR, some documents are still in the physical paper since it requires the patients' signature, and most of them do not have a DS. Therefore, patients should wait for an administrative review of all paper documents before leaving the hospital. After 2017, Brazilian regulations started to accept the encrypted digital signature as a valid alternative to overcome this problem. The leading nurse DS in the EHR can now attest that all the papers are in order. We implemented this solution for all healthcare personnel and used it as the primary intervention in this project.

This intervention had an immediate and sustained effect, reducing 3.6 h of hospital bed turnover. In 2013, The Advisory Board Company (www.advisory.com) estimated a reduction of 0.25 days in the duration of hospital stay for an institution of 600 beds would provide an extra 25 beds available in 24 h. This amount of beds is what we should expect to accommodate the unscheduled hospital admissions of HOC patients.

Even though the reduction in time to discharge was immediate, the same did not occur to the number of patients directed to the ED. There could be several explanations for this finding. First, an institutional culture (a "reflex") to direct patients to the ED instead of looking for an available bed on the floor. Second, each specialty has some designated beds for their use in our institution. The "new" bed that the intervention provided was not always that of the specialty for patients needing in-hospital admission. We corrected that by implementing an allocation beds central dashboard and guaranteed the teams that "their" beds would be available again in 24 h if they "lend" them to the specialty in need [9]. Third, some patients require intensive care units and positive or negative isolation beds (for cases of tuberculosis or immune depressed conditions, respectively), which are more scarce in the institution. Finally, external factors such as the remission of other hospitals to our ED could influence the outcome. Unfortunately, we could not measure those confounders.

Besides the impact of expediting the patient discharge, another positive result was that the patients left the hospital earlier (15h00 to 16h00). Our patients come from small cities and depend on working-hour public transportation to go home after discharge. If the discharge occurs after 17h00, there is no public transportation, the patient has to remain in the hospital up to the following day, and we could not use the bed for another patient waiting in the ED. This problem is aggravated during weekends when this public transportation is not available. We attributed the bimodal distribution before the intervention to the nurse duty shift. The shift to the left and assuming a normal time delay distribution for the patients leaving the hospital after the intervention corroborates this. Other authors showed similar findings [10, 11].

Our approach to avoiding boarder by expediting discharge after medical order is a pull forward strategy since the available bed will "pull" patients waiting for admission to the floor. Another process recently reviewed in the literature is a push-forward one, represented by the transfer of patients to a lounge on the floor, so the floor staff will try to guarantee an available bed [12]. This push-forward strategy still lacks definitive evidence of effectiveness. Our pull-forward approach seems more logical than the push-forward since it addresses the floor staff's needs instead of putting pressure on them. We acknowledged that our strategy still requires further studies for a definitive cause-effect relationship. Besides, one should consider differences among institutions, including their culture, while searching for solutions to avoid boarders in ED.

Our findings follow others. Mustafa et al. (2016) demonstrated that the patients directed to ED from HOC due to an unscheduled admission have a solid association with crowding. Furthermore, patients with delayed discharge occupied up to 15% of the hospital beds [2]. Majeed MU et al. (2012) presented similar results, emphasizing elderly patients' role in this situation [13]. Silva et al. (2014) presented similar data in Brazil. They pointed to teaching hospitals such as ours that could suffer from a more significant delay for patients effectively leaving the hospital due to the learning curve of the health care professional in training [14].

Ideally, following patients' flow in a thorough output model, including more active measures such as a dedicated person, would bring more immediate results in reducing boarders [15]. Nevertheless, our institution has limited resources, and the solution implemented is very cost-effective. After the present study's findings, as previously mentioned, we implemented a dedicated central follow-up patient flow, including a Hospital Medicine professional that can intervene proactively to reduce LOS [16, 17]. Newer strategies such as discrete events simulation could work with BPM to evaluate an intervention's impact before studying it in the field [18].

## Data Availability

We extracted data from our institutional Electronic Health Record. If there is interest in accessing the data used in this study, the authors will have to consult the Ethical Committee prior to granting access.

## Abbreviations

BPM: Business Process Management
DS: Digital Signature
ED: Emergency Department
HER: Electronic Health Record
HOC: Hospital Out Clinic
ITS: Interrupted Time-Series Analysis
LOS: Length of Stay
